# Alteration of Testosterone Levels Changes Brain Wave Activity Patterns and Induces Aggressive Behavior in Rats

**DOI:** 10.3389/fendo.2019.00654

**Published:** 2019-09-24

**Authors:** Daniel Pantoja Estumano, Luan Oliveira Ferreira, Paulo Augusto Lima Bezerra, Maria Clara Pinheiro da Silva, Giovanna Coutinho Jardim, George Francisco Souza Santos, Kayo Silva Gustavo, Bruna Gerrits Mattos, Jorge Amando Batista Ramos, Vanessa Jóia de Mello, Edmar Tavares da Costa, Dielly Catrina Favacho Lopes, Moisés Hamoy

**Affiliations:** ^1^Laboratory of Pharmacology and Toxicology of Natural Products, Institute Biological Science, Federal University of Pará, Belém, Brazil; ^2^Laboratory of Experimental Neuropathology, João de Barros Barreto University Hospital, Federal University of Pará, Belém, Brazil; ^3^Laboratory of Human Cytogenetic, Institute Biological Science, Federal University of Pará, Belém, Brazil

**Keywords:** testosterone, brain waves, behavior, anabolic, supplementation, electrocorticographic

## Abstract

Testosterone is responsible for several changes in the brain, including behavioral and emotional responses, memory, and cognition. Given this, we investigated changes in the brain wave profile caused by supplementation with exogenous testosterone in both castrated and non-castrated rats. We also investigated the serum testosterone levels, renal and hepatic function, and the lipid and behavioral profiles. We found changes in the spectral wave power in both groups (castrated and non-castrated animals) supplemented with exogenous testosterone, consistent with an aggressive/hostile profile. These changes were observed in the electrocorticographic evaluation associated with increased power in low-frequency (delta and theta) and high-frequency (beta and gamma) activity in the supplemented animals. The castrated animals presented a significant decrease of wave power in the alpha frequency. This correlated with a decrease of the performance of the animals in the elevated plus-maze evaluation, given that the alpha wave is linked to the execution and visualization of motor processes. In the behavioral evaluation, the castrated animals presented a reduced permanence time in the elevated-plus maze, although this was prevented by the supplementation of testosterone. Testosterone supplementation induced aggressive behavior in non-castrated animals, but not in castrated ones. Supplemented animals had significantly elevated serum testosterone levels, while their urea levels were significantly lower, but without clinical significance. Our data indicate that testosterone supplementation in non-castrated rats, but not in castrated ones, causes electrocorticographic changes that could be associated with more aggressive and hostile behavior, in addition to indicating a potential for personality disorder. However, further studies are required to elucidate the cellular and molecular changes caused by acute testosterone supplementation.

## Introduction

Testosterone is the major anabolic androgenic steroid (AAS) hormone, which plays a key role in brain development (1). This hormone is predominant in males and has numerous physiological roles, acting in both the central nervous system (CNS) and peripheral tissue (2). The effects of testosterone on the brain are crucial to development and sexual behavior, and are responsible for the differences between the sexes (3).

Testosterone acts as a neurosteroid in the neurons, where it may induce changes at the cellular level, affecting behavior, memory, cognition, and emotion (4–6). Some of the neuromodulatory effects of testosterone in the CNS have been studied previously (7, 8), mainly because it causes glial modulation (9). The long-term effects of this hormone on brain development also involve epigenetic modifications (10).

Testosterone levels increase substantially in humans (young people and athletes) that use high doses of AASs, mainly testosterone derivatives, to induce muscle hypertrophy (11), or to improve performance or esthetics (12, 13). The abusive supplementation of AASs has been associated with a large number of collateral psychiatric effects (panic, depression/anxiety, and obsessive-compulsive disorders or hyperactivity). This may result in negative behavioral changes, including some sexual disturbances, given that it also acts on the pre-optic area, a region of the brain responsible for sexual behavior (14). There is evidence that AASs users present more hostile behavior, as well as symptoms of somatic disorders, depression, and anxiety, together with paranoia (15), which could even lead to suicide, in some cases (16, 17). These mechanisms have not yet been fully elucidated, especially in terms of their actions in the brain.

In experimental models, testosterone may be excitatory (18) or inhibitory (19), indicating that it has been converted into metabolites with different biological activities in the brain. These metabolites may increase the discharge threshold in the CNS, exerting inhibitory or excitatory actions, possibly by modulating the GABA-A or glutamate receptors (20). These convulsive discharges may affect the hypothalamic-pituitary axis and cause hormones to be released at inappropriate times, leading to hormonal and behavioral alterations. For these reasons, the effect of testosterone on cerebral excitability demands further investigation.

In this context, the present study investigated the effects of testosterone supplementation in non-castrated and castrated male rats, through the evaluation of the electrocorticographic (ECoG) activity, and the behavioral, biochemical, and hormonal changes provoked by the treatment in these animals.

## Materials and Methods

### Animals

Seventy-eight adult male Wistar rats (280 ± 20 g) were obtained from the Central Animal Facility of the Federal University of Pará. All the animals were housed under controlled conditions, with a temperature of ~22°C and a 12/12 h light-dark cycle, with food and water available *ad libitum*. All experimental procedures were conducted in accordance with the principles of laboratory animal care (21), and were approved by the Ethics Committee on Experiments in Animals of the Federal University of Pará (CEUA no. 7338220818). All necessary procedures were conducted to prevent animal suffering and distress.

### Chemicals

The anesthetic ketamine was purchased from König (Santana de Parnaíba, SP, Brazil) and the anesthetic xylazine from Vallée (Montes Claros, MG, Brazil), while the local anesthetic lidocaine was obtained from Hipolabor (Sabará, MG, Brazil). Testosterone esters were obtained from the commercial formulation Durateston® produced by the Organon Laboratory of Brazil (São Paulo, SP, Brazil). Durateston® is widely used as a treatment for some clinical conditions (22) and by athletes to improve their performance (12, 13).

### Experimental Design

**Figure 1:**
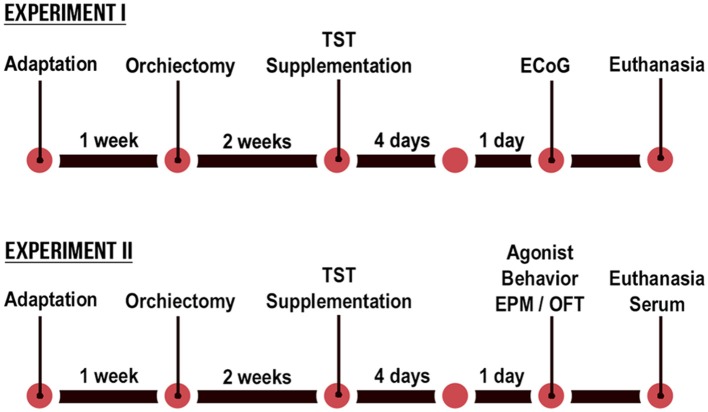
Schematic diagram of the timeline of the testosterone supplementation in non-castrated and castrated rats.

The present study was based on two separate experiments, with an identical design. The only difference in the experimental designs was in the final evaluation, with that of Experiment I being based on ECoG recordings, whereas in Experiment II the animals were assessed in an elevated plus-maze (EPM), open field test (OFT), agonistic encounters with an intruder male, and biochemical and hormonal analyses (Figure 1). The orchiectomized and sham animals (12 weeks old) were operated 2 weeks before the start of the experimental protocol to reduce the levels of circulating TST from testicular secretions in the castrated animals. Fourteen weeks old, intact and castrated male rats were randomly divided into vehicle-treated [non-castrated (NC) and castrated (C)] groups and testosterone-supplemented [non-castrated + testosterone (NC + TST) and castrated + testosterone (C + TST)].

The testosterone (Durateston®) dosage was determined by the results of a pilot experiment. Doses of 15, 20, 25, and 30 mg/kg (23, 24) were tested for 4 days, once a day. On the fifth day, the potential for aggressive behavior in the animals was tested by the presentation of an intruder male for 10 min. At a dose of 25 mg/kg (via intraperitoneal, i.p.), the animals exhibited aggressive behavior after 2 min of contact with the intruder, so this dose was chosen for the experiments. The vehicle groups received a 0.9% physiological solution at an equivalent volume (mL) to weight (kg) ratio.

The animals were assessed 24 h after the final injection (Figure 1), according to the experiment, with the collection of electrocorticographic records in the case of Experiment I and behavioral and biochemical analyses in the case of Experiment II. The body weight of each animal was obtained daily in order to calculate the injection volume required to administer the desired dose.

### Orchiectomy

The animals to be castrated were anesthetized with ketamine hydrochloride (80 mg/kg) and xylazine hydrochloride (10 mg/kg). The scrotum was shaved using a sterile razor and a bilateral orchiectomy was performed using the scrotal approach. An absorbable suture was used to ligate the blood vessels and the vas deferens. Both the testis and the epididymal fat pad were removed. The skin incision was closed using a non-absorbable suture and the rats were returned to their cages once they regained consciousness. A dose of dipyrone (25 mg/kg, i.p.) was administered for pain relief, and the antibiotic amoxicillin [30 mg/kg, via intramuscular (i.m.)] was also administered for 2 days. The non-castrated groups were sham operated.

### Electrocorticographic Recordings and Data Analyses (Experiment I)

The animals were anesthetized with ketamine hydrochloride (80 mg/kg, i.p.) and xylazine hydrochloride (10 mg/kg, i.p). After the abolishment of the corneal reflex, the animals were placed in a stereotaxic apparatus. After surgical procedures to expose the skull, two bilateral holes were drilled into the skull with a dental drill. Stainless steel electrodes (exposed tip 1.0 mm in diameter) were then placed on the dura mater above the pre-frontal cortex at the bregma coordinates−0.96 mm and ± 1.0 mm lateral (25). A screw was fixed to the occipital bone, and the electrodes were fixed with dental acrylic cement using the screw as the base and grounding for the ECoG recordings.

Following surgery, the animals were kept in individual cages. Seven days after surgery, the electrodes were connected to a digital data acquisition system composed of a high impedance amplifier (Grass Technologies, P511), an oscilloscope (Protek, 6510), and a data acquisition and digitalization board (National Instruments, Austin, TX). Data were collected continuously at 1 kHz, at a low pass of 3 kHz and high pass of 0.3 Hz. During the recording sessions, the animals were confined to a restricted space in acrylic boxes (20 × 45 × 15 cm). The ECoG followed a standard protocol in all treatments, with 10 min of accommodation, followed by 300 s of ECoG recording.

Offline analyses were run using a tool built in the Python programming language (version 2.7), with “Numpy” and “Scipy” libraries being used for the mathematical processing and a “matplolib” library to obtain the graphs and plots. A graphic interface was developed using the PyQt4 library. Spectrograms were calculated using a 256-point Hamming window (256/1,000 s). Each frame of the power spectral density (PSD) was generated with an overlap of 128 points per window. For each frame, the PSD was calculated by Welch's average periodogram method. The frequency histograms were obtained by calculating the PSD of the signal using the 256-point Hamming window without overlap, which yielded a resolution of 1 Hz per bin. Each wave displayed in the PSD is an average from a given set of experiments. The PSD was calculated for each group, and the means were shown in individual bins. These analyses were run at a frequency of up to 50 Hz, and split into Delta (1–4 Hz), Theta (4–8 Hz), Alpha (8–12 Hz), Beta (12–28 Hz), and Gamma (30–40 Hz) bands for the interpretation in accordance with previous studies (25, 26).

### Behavioral Analysis (Experiment II)

Open-field test (OFT) was conducted on the experimental animals to determine their locomotor activity. The OFT apparatus consists of a square-based box (100 × 100 cm, 50 cm in height) open at the top. Each animal was placed at the center of the arena (box floor) and allowed to explore for 5 min, after which, it was removed. The distance moved, time spent moving, and mean speed were all measured.

Anxiolytic activity was analyzed in an elevated plus-maze (EPM) test that consisted of two open arms (50 × 10 × 0.5 cm) arranged in opposition to two closed arms (50 × 10 × 40 cm) elevated 50 cm above the floor. In this procedure, three parameters were determined—the total time the animal spent on the test (up to 300 s), the percentage of time spent in the open arms ([time in the open arms/ (time in the open arms + time in the closed arms)] × 100), and the percentage of entries into the open arms ([entries in the open arms/(entries in the open arms + entries in the closed arms)] × 100). Both tests were conducted in the daylight, between 08:00 a.m. and 01:00 p.m., and the data were analyzed using the ANY-Maze software (Stoelting Co, Wheat Lane, Wood Dale, IL, USA).

The rats were also tested for agonistic behavior using the resident-intruder paradigm (27). Resident animals were first removed from their home cages, and, after a 5 min acclimation period, an age- and weight-matched male intruder was placed in the home cage simultaneously with the resident rat (NC, C, NC + TST, or C + TST). Behavioral tests of 10 min duration were then conducted and the number of aggressive behavior was counted. During each test, the number (n) and duration (s) of dominance (contact) events (sniffing or approaching), aggressive events (offensive posturing, and the number of attacks and bites), and the total number of dominant + aggressive behaviors directed toward the intruder were quantified. The number (n) and duration (s) of submissive behaviors (defensive posturing, walking with tail upright, and escape dashes) were also determined. The intruders were used in more than one behavioral test. All the rats were tested during the first 4 h of the dark cycle under dimmed red light conditions to control for the potential influence of circadian variables on behavioral responses.

### Biochemical and Hormonal Analyses (Experiment II)

For the biochemical procedures, serum was collected for the analysis of the levels of urea, creatinine, cholesterol, high-density lipoprotein (HDL), low-density lipoprotein (LDL), triglyceride, glutamate oxaloacetate transaminase (GOT), glutamate pyruvate transaminase (GPT), and testosterone. Plasma testosterone was measured by chemiluminescent assay (Abbott Lab, Chicago, Illinois, USA). Serum triglyceride, LDL, and cholesterol levels were measured using the colorimetric assay kit (Labtest, MG, Brazil). The commercial direct oxidase assay kit (Labtest, MG, Brazil) was used to measure total HDL levels. The plasma GOT and GPT levels were also measured using the ultraviolet assay kit, without the pyridoxal phosphate (Labtest, MG, Brazil). The colorimetric technique was used to determine urea levels (urease) and plasma creatinine was assayed using the kinetic absorbance method (Labtest, MG, BRA). All the readings were taken using a Mindray BS-200 apparatus (Shenzhen, Nanshan, China).

### Statistical Analyses

The normality and homogeneity of the variances of the data were verified using Kolmogorov-Smirnov and Levene's tests, respectively. Data are presented as means ± standard deviation (SD) and the *F* and *p* values are included, where pertinent. A *p* < 0.05 significance level was considered for all analyses. Between-group comparisons were based on a two-way ANOVA followed by Tukey's test for multiple comparisons. Statistical analyses for the identification and removal of the outliers were run in GraphPad Prism, version 8 (Graph-Pad Software Inc., San Diego, CA, USA).

## Results

### Testosterone Supplementation Changes ECoG Pattern in Rats

**Figure 2:**
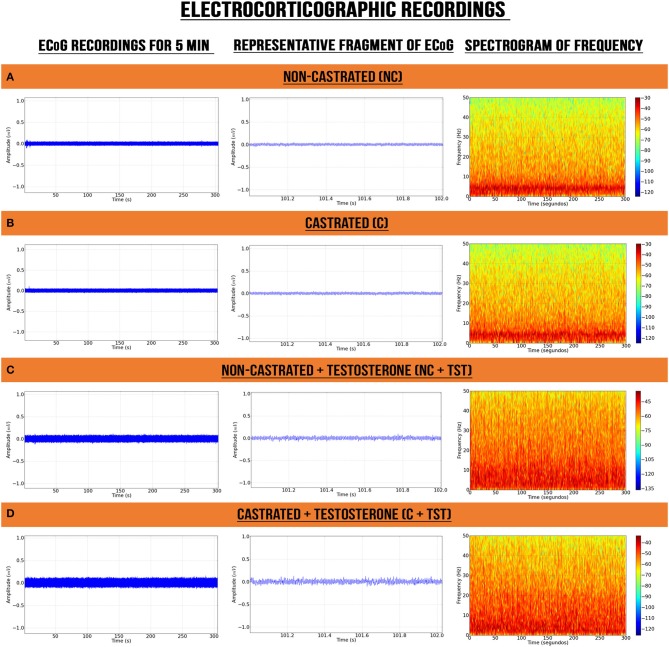
Electrocorticographic recordings of intact (non-castrated) and castrated animals with/without testosterone supplementation. **(A)** Non-castrated group. **(B)** Castrated group. **(C)** Non-castrated group with testosterone supplementation. **(D)** Castrated group with testosterone supplementation.

The ECoG recordings from the vehicle groups of non-castrated (NC) and castrated (C) rats presented certain similarities in their electrocorticographic readings, such as a higher energy intensity below 10 Hz (Figures 2A,B). By contrast, the groups with testosterone supplementation (NC + TST and C + TST) presented power distributions oscillating below 40 Hz with alterations in the electrocorticographic tracings of all brain waves (Figures 2C,D).

### Testosterone Supplementation Alters Cerebral Waves Amplitude

**Figure 3:**
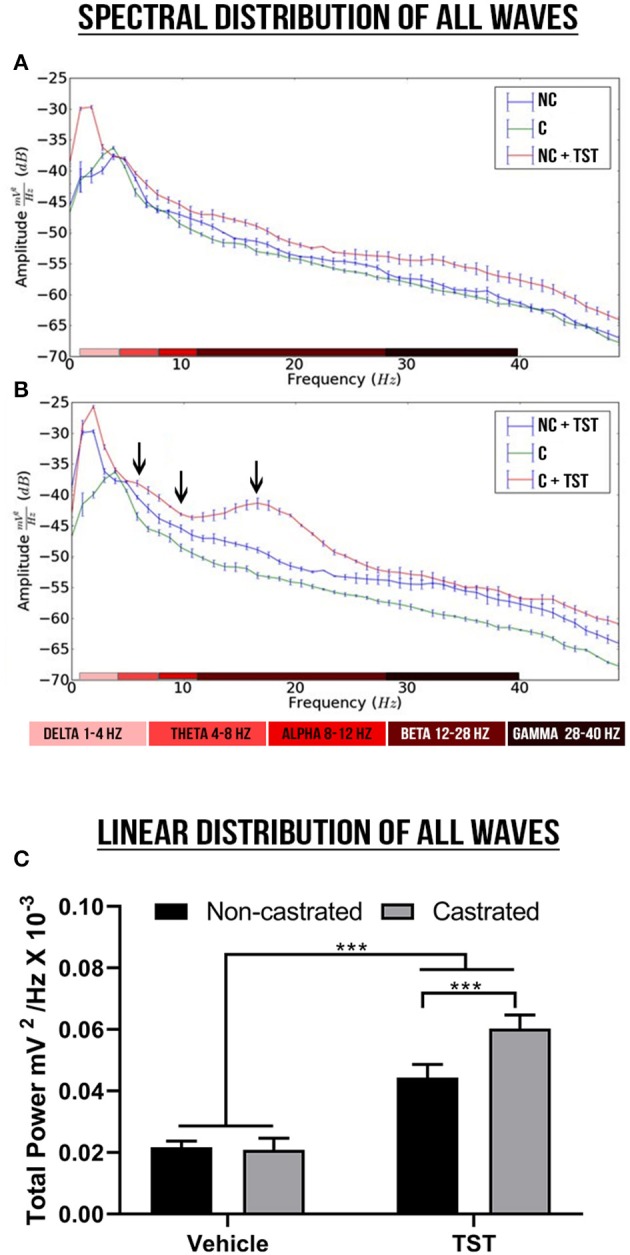
Spectral power and linear frequency distributions of intact and castrated animals with/without testosterone supplementation. **(A,B)** Represent the spectral power distributions of the delta, theta, alpha, beta, and gamma cerebral waves between groups, while the arrows indicate the oscillations. **(C)** Represents the linear frequency distribution between groups of power up to 40 Hz. The data are expressed as means ±SD (*n* = 9 animals/group; ***p < 0.001). (NC, non-castrated; C, castrated; NC + TST, non-castrated + testosterone; C + TST, castrated + testosterone).

The decomposition of the spectral power distribution revealed greater amplitude in the delta, theta, alpha, beta, and gamma waves in the groups with testosterone supplementation than in those without supplementation (Figures 3A,B). The supplemented castrated group (C + TST) presented the greatest oscillations in amplitude, highlighting alterations in the theta, alpha, and beta waves (see Figure 3B).

In the linear frequency distribution analysis up to 40 Hz, there was a significant interaction between the effects of testosterone supplementation and castration on the total wave power [*F*_(1, 32)_ = 45.96; *p* < 0.0001], although no difference was observed in the main effect on the total power between the non-castrated and castrated vehicle groups (NC: 0.02171 ± 0.001989 mV2/Hz × 10^−3^; C: 0.02086 ± 0.003785 mV2/Hz × 10^−3^; *p* = 0.9617). However, testosterone supplementation increased total wave power significantly more in the castrated animals (0.060031 ± 0.004370 mV2/Hz × 10^−3^) than the intact animals (0.04436 ± 0.004232 mV2/Hz × 10^−3^; *p* < 0.0001) and in comparison with the vehicle groups (*p* < 0.0001; Figure 3C).

**Figure 4:**
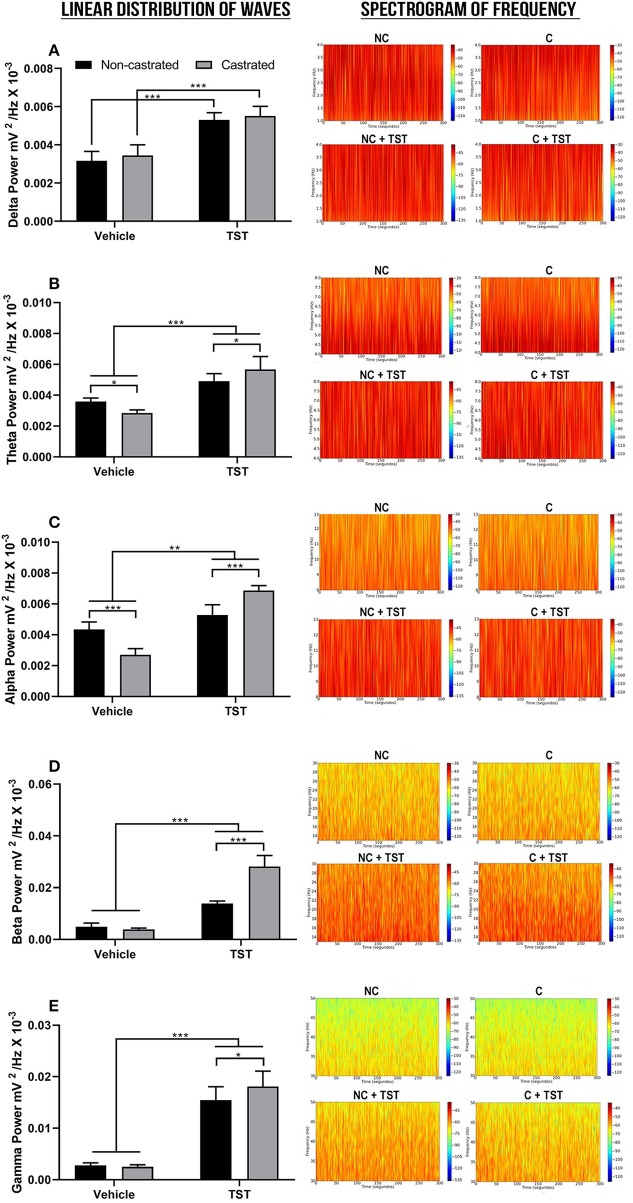
Decomposition of the cerebral waves. **(A)** Linear frequency distribution (left) and spectrogram of the frequencies (right) of the delta waves. **(B)** Linear frequency distribution (left) and spectrogram of the frequencies (right) of theta waves. **(C)** Linear frequency distribution (left) and spectrogram of the frequencies (right) of the alpha waves. **(D)** Linear frequency distribution (left) and spectrogram of the frequencies (right) of the beta waves. **(E)** Linear frequency distribution (left) and spectrogram of the frequencies (right) of the gamma waves. The data are expressed as means ±SD. (*n* = 9 animals/group; *p < 0.05, **p < 0.01, and ***p < 0.001). (TST, testosterone; NC, non-castrated; C, castrated; NC + TST, non-castrated + testosterone; C + TST, castrated + testosterone).

The decomposition of the cerebral waves was also analyzed. In the case of the delta wave (1–4 Hz), significant differences were only found in relation to testosterone supplementation [*F*_(1, 2)_ = 166.9; *p* < 0.0001]. Specifically, TST supplementation resulted in a significant increase in delta wave oscillations in comparison with the vehicle group (NC: 0.003162 ± 0.0004954 mV2/Hz × 10^−3^ vs. NC + TST: 0.005302 ± 0.0003827 mV2/Hz × 10^−3^; *p* < 0.0001; Figure 4A). Similarly, delta power also increased significantly in the treated-castrated rats, in comparison with the vehicle-castrated animals (C: 0.003444 ± 0.0005569 mV2/Hz × 10^−3^ vs. C + TST: 0.005512 ± 0.0005030 mV2/Hz × 10^−3^; *p* < 0.0001).

In the case of the theta wave frequency (4–8 Hz), all the treatments (testosterone supplementation and castration) were significantly different from one another [*F*_(1, 32)_ = 19.66; *p* = 0.0001]. Non-castrated animals had a mean theta power of 0.003592 ± 0.0002202 mV2/Hz × 10^−3^. The absence of testosterone decreased the theta power in the castrated group (0.002843 ± 0.0002059 mV2/Hz × 10^−3^) when compared to the NC group (*p* = 0.0195). Testosterone supplementation increased theta power much more in the castrated animals (0.005665 ± 0.0008419 mV2/Hz × 10^−3^), however, than in the non-castrated ones (0.004904 ± 0.0004922 mV2/Hz × 10^−3^; *p* = 0.0172) and both groups in comparison with the NC and C groups (*p* < 0.0001; Figure 4B).

As for the theta waves, all the groups were significantly different (interactions) from one another [*F*_(1, 32)_ = 102.3; *p* < 0.0001] in the alpha waves (8–12 Hz). The castrated animals had the lowest alpha power of all the groups, with a mean of 0.002703 ± 0.0003969 mV2/Hz × 10^−3^ (*p* < 0.0001 for all comparisons). In the groups supplemented with testosterone, the greatest increase was recorded in the castrated rats (0.006868 ± 0.0001051 mV2/Hz × 10^−3^), higher than that recorded in the non-castrated rats (0.005276 ± 0.0006682 mV2/Hz × 10^−3^; *p* < 0.0001), and both groups greater than that recorded in the non-castrated vehicle animals (NC: 0.004356 ± 0.0004722 mV2/Hz × 10^−3^; *p* < 0.01; Figure 4C).

In the case of the beta wave frequency (12–28 Hz), there was a significant interaction between the effects of testosterone supplementation and castration [*F*_(1, 32)_ = 99.61; *p* < 0.0001]. The NC (0.004899 ± 0.001419 mV2/Hz × 10^−3^) and C groups (0.003894 ± 0.0004763 mV2/Hz × 10^−3^) did not vary significantly (*p* = 0.7916). However, the groups supplemented with testosterone presented an increase in beta wave oscillation, with the highest response being recorded in the castrated rats (NC + TST: 0.01386 ± 0.0009710 mV2/Hz × 10^−3^ vs. C + TST: 0.02819 ± 0.004250 mV2/Hz × 10^−3^; *p* < 0.0001), and both groups were significantly higher than the vehicle groups (*p* < 0.0001; Figure 4D). Overall, the greatest oscillations caused by testosterone supplementation were recorded at this wave frequency.

As in the case of the delta and beta waves, no difference was found between the NC (0.002780 ± 0.0005126 mV2/Hz × 10^−3^) and C groups (0.002524 ± 0.0003861 mV2/Hz × 10^−3^; *p* = 0.9932) at the gamma wave frequency (28–40 Hz), although there was a significant interaction between both the treatments tested [testosterone supplementation and castration; *F*_(1, 32)_ = 4.572; *p* = 0.0402]. In addition, testosterone supplementation altered the gamma wave frequencies in both groups, with the greatest increase being recorded in the castrated animals (NC + TST: 0.01545 ± 0.002624 mV2/Hz × 10^−3^ vs. C + TST: 0.01808 ± 0.003011 mV2/Hz × 10^−3^; *p* = 0.0450) and both groups in comparison with the vehicle groups (*p* < 0.0001; Figure 4E).

### Testosterone Supplementation Altered Physiological Parameters Such as Serum Testosterone Levels and Urea, but Did Not Change Lipid Profile, Creatinine, or Hepatic Enzymes

**Figure 5:**
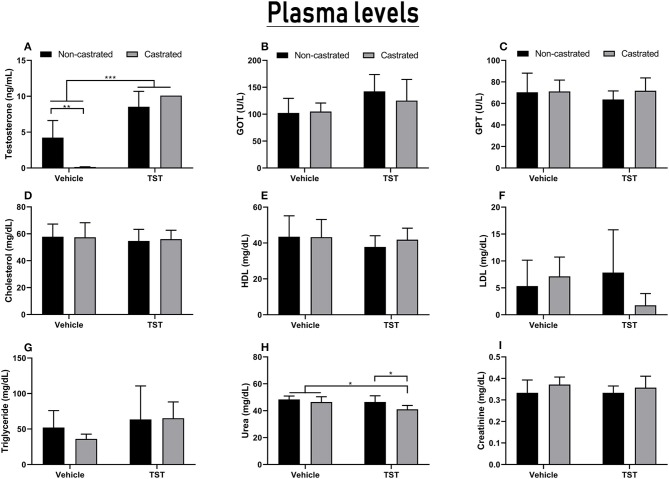
Results of the hormonal and biochemical analyses of the castrated and intact animals with/without testosterone supplementation. **(A)** Serum testosterone levels. **(B,C)** Liver function based on GOT and GPT enzymes levels. **(D–G)** Lipid profiles including cholesterol, LDL, HDL, and triglyceride levels. **(H,I)** Renal function based on urea and creatinine levels. The data are expressed as means ±SD (*n* = 6–8 animals/group, *p < 0.05, **p < 0.01 and ***p < 0.001). (TST, testosterone).

Testosterone levels interacted significantly with both treatments tested [*F*_(1, 20)_ = 18.64; *p* = 0.0003]. The mean testosterone level recorded in the intact (non-castrated) animals observed in the experiments presented here was 4.24 ± 2.38 ng/mL. The orchiectomy was effective for the reduction of serum testosterone levels in the castrated animals (0.14 ± 0.03 ng/mL) in comparison with the intact animals (*p* = 0.0014), indicating that bilateral orchiectomy is effective as a model of testosterone deprivation. However, rats supplemented with 25 mg/kg of testosterone for 4 days presented a significant increase in serum testosterone levels. Thus, non-castrated (8.54 ± 2.14 ng/mL) and castrated animals (>10.09 ng/mL) with TST supplementation presented serum testosterone levels higher than unsupplemented intact and castrated animals (*p* < 0.001). It is important to note that the C + TST and NC + TST groups had testosterone levels higher than the detectable limit of the assay (Figure 5A).

The biochemical analysis showed that TST supplementation for 4 days did not change the lipid profile or liver enzymes of the treated animals (Figures 5B–G).

Although the combined effects of castration and testosterone supplementation could not be differentiated, the two factors did have significant effects when considered individually, i.e., testosterone supplementation [*F*_(1, 23)_ = 6.896; *p* = 0.0151] and castration [*F*_(1, 23)_ = 7.170; *p* = 0.0134]. In particular, significantly lower levels of urea were observed in the C + TST group (41.00 ± 2.9 ng/dL) in comparison with the C (46.43 ± 3.95 mg/dL; *p* = 0.0344) and NC + TST groups (46.50 ± 4.59 ng/dL; *p* = 0.0414; Figure 5H). No alteration was observed in the creatinine levels (Figure 5I).

### Behavioral Effects of Testosterone Supplementation

**Figure 6:**
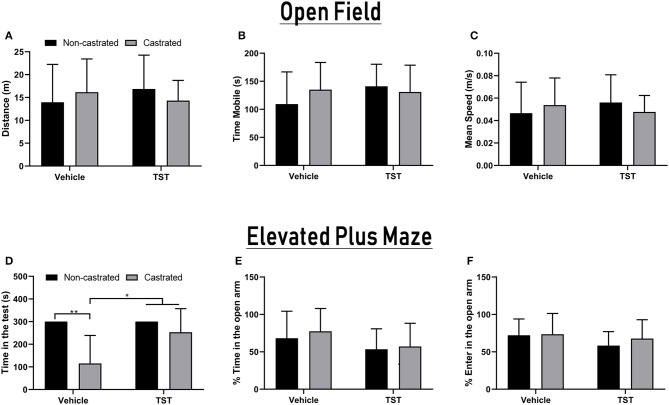
Behavioral parameters in intact and castrated animals with/without testosterone supplementation. Open Field Test: **(A)** Total distance traveled, **(B)** time mobile, and **(C)** mean speed of movement. Elevated Plus-Maze test: **(D)** total time in the apparatus, **(E)** percentage of time in the open arms, and **(F)** percentage of entries into the open arms. The data are expressed as means ±SD (*n* = 9–12 animals/group, *p < 0.05 and **p < 0.01). (TST, testosterone).

The OFT and EPM tests were applied to measure the influence of castration and/or testosterone supplementation on the rats' behavior. In the case of the OFT analysis, no significant variation was observed in relation to any of the parameters analyzed (Figures 6A–C). In the EPM, there was significant interaction between the effects of testosterone supplementation and castration on the time spent in the apparatus [*F*_(1, 33)_ = 5.362; *p* = 0.0269]. The animals of the castrated group (115.22 ± 123.5 s) spent significantly less time in the apparatus than those of the NC (300.00 ± 0.00 s; *p* = 0.0008), NC + TST (300.00 ± 0.00 s; *p* = 0.0005), and C + TST groups (253.28 ± 104.02 s; *p* = 0.0048; Figure 6D). No significant variation was found in the percentage of time spent in the open arm, nor in the percentage of entries into the open arm (Figures 6E,F).

**Figure 7:**
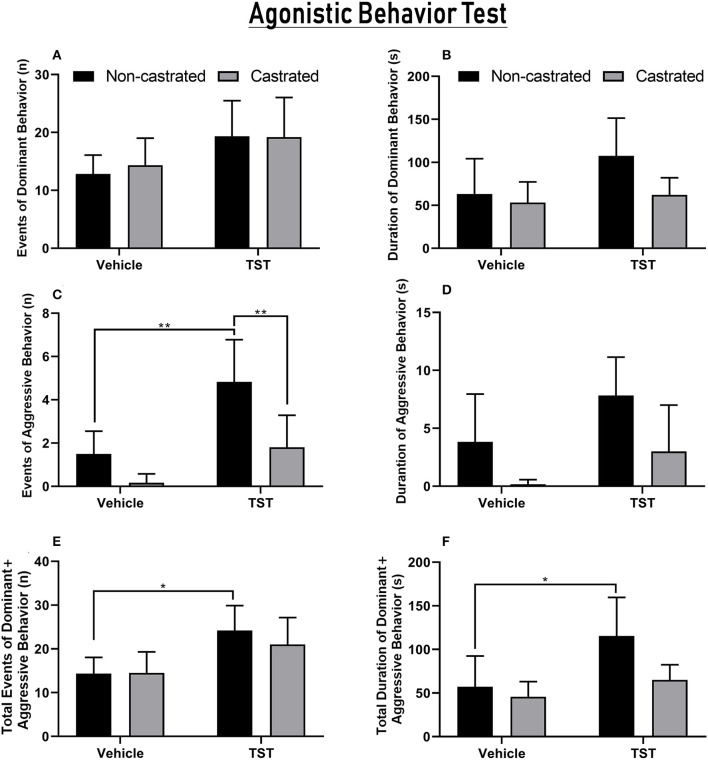
Agonistic behaviors recorded in the tests using a resident–intruder paradigm. **(A)** Number of events of dominant behavior (contact events), **(B)** duration of dominant behavior, **(C)** number of events of aggressive behavior, **(D)** duration of aggressive behavior, **(E)** number of total events of dominant + aggressive behaviors toward the intruder (contact time, offensive posturing, and the number of attacks and bites), **(F)** total duration of dominant + aggressive behaviors toward the intruder. The data are expressed as means ±SD (*n* = 6–7 animals/group, *p < 0.05 and **p < 0.01). (TST, testosterone).

Agonistic behaviors are shown in Figure 7. No significance variation was found in the number or duration of dominance events among the intact, castration, vehicle, and testosterone supplementation groups (Figures 7A,B).

Significant variation was found in the characteristics of the aggressive events in relation to both testosterone supplementation [*F*_(1, 19)_ = 19.71; *p* = 0.0003] and castration [*F*_(1, 19)_ = 15.24; *p* = 0.0010]. The number of aggressive events increased significantly in non-castrated rats after testosterone supplementation (4.83 ± 1.94), compared with the non-castrated vehicle (1.5 ± 1.05; *p* = 0.0019) and castrated + testosterone groups (1.8 ± 1.48; *p* = 0.0068). In the case of the duration of aggressive events, while significant variation was found on response to the main effects, this was not sustained in the *post-hoc* test (Figures 7C,D).

In the case of the total number of events of dominant/aggressive behavior, a significant difference was only found in relation to testosterone supplementation [*F*_(1, 19)_ = 14.49; *p* = 0.0012]. Specifically, the total number of events increased significantly in non-castrated rats after testosterone supplementation (24.17 ± 5.7) in comparison with the non-castrated vehicle group (14.33 ± 3.72; *p* = 0.0173). Testosterone supplementation also had a significant effect on the total duration of dominant/aggressive behavior [*F*_(1, 18)_ = 6.350; *p* = 0.0214]. The NC + TST group (115.33 ± 44.18 s) presented the greatest increase in the duration of dominant behavior in comparison with the vehicle groups (NC: 57.2 ± 35.21 s; *p* = 0.0376; Figures 7E,F). No significant variation was found in the other comparisons.

No significant variation in submissive behavior was observed between groups with/without testosterone supplementation or between the non-castrated/castrated groups (data not shown).

## Discussion

The organic release of steroid hormones, such as testosterone, follows a pulsatile circadian pattern that is managed by a central clock located in the suprachiasmatic nucleus of the hypothalamus. This system receives environmental light cues from the retinas and adjusts the release of hormones to the daytime cycle (28). In this context, gonadectomy changes the period of circadian behavioral rhythms, and decreases overall brain activity, an effect recuperated by testosterone replacement (29). However, some studies have shown that a single injection of testosterone was sufficient to increase the functional connectivity between the cortico-cortical circuits, contributing to both inter- and intra-hemispheric functional communication (30), and it may also modulate physiology, cognition, and behavior, promoting competition and aggression (31).

From this perspective, the excessive use of AASs by athletes and non-athletes to enhance performance or physical appearance appears to be common (13), even though it is associated with a large number of physical and psychological complications, such as hypertension, myocardial hypertrophy, and psychiatric or behavioral symptoms, such as aggressiveness and irritability (12, 32, 33). These symptoms may be associated with changes in the brain connections responsible for emotional processing in the hippocampal and pre-frontal-cortical areas, for example (34).

Experimental studies have found evidence that androgen hormones affect the size and number of neurons, and the organization of synapses and the neurotransmitter system, and that these mechanisms are mediated by genomic pathways (35, 36). The effects of testosterone on the brain are not fully known, but abrupt variations in hormone concentrations, as in acute supplementation, may change cerebral connections (37) and may be able to alter social and sexual behavior by modifying functions in the hypothalamic and cortical areas (30).

In this context, the results of the present study of post-pubescent castrated and non-castrated male rats confirmed the hypothesis that testosterone supplementation is associated with a change in ECoG patterns, in particular, a marked increase in brain activity. From this perspective, the changes observed in the power patterns of the decomposed waves were consistent with those of previous studies. In the studies of Juárez et al. (38) and Poblano et al. (39), testosterone supplementation increased the relative power of the delta and theta waves in the frontal and temporal cortices and these changes may be related to changes in the organization of the brain. Schutter et al. (30) also showed that only one dose of exogenous testosterone was sufficient to increase the trigger force of the delta waves in the interhemispheric readings (left pre-frontal and right parietal cortices), indicating changes in emotional processing. This is consistent with the symptoms found in individuals that use AASs, including mood changes, insomnia, and anxiety (12), which suggests that testosterone supplementation, can pre-dispose individuals to depression-like symptoms.

The results of the present study also showed increased amplitude of shots in animals with testosterone supplementation in the ECoG (Figure 2). There was also an increase in the wave power of all frequency bands, in particular in the beta wave oscillations. In this context, the increase of beta waves in the frontoparietal region may have been related to the more aggressive and hostile behavior observed in these animals (40–42). This activity is thought to be the result of cortical/cortical and thalamo/cortical interactions, which may indicate cortical hyperexcitability (43).

A similar increase in beta wave activity has been described in previous studies of violent individuals with varying degrees of psychopathy (44, 45). Similar findings were also obtained in subjects with moderate intermittent explosive disorder (46), in impulsive individuals (47), and in children with hyperactivity (48). Furthermore, individuals with antisocial personality disorder presented not only an increase in beta wave activity, but also in all other types of brain wave (49). The behavioral and ECoG patterns observed in the present study indicate that testosterone supplementation may be related to a broader spectrum of behavior disorders than a simple increase in impulsive actions and behavioral disinhibition.

Castrated rats treated with testosterone presented the highest total wave power and relative power in almost all waves, except the delta waves, in comparison with supplemented non-castrated rats. This may be accounted for by the fact that androgens often regulate the expression of their receptors in direct proportion to the difference between their density and serum levels (50, 51). In the present study, however, the gonadectomized males underwent a period of hormonal deprivation, which may have made their receptors more sensitive to testosterone supplementation, which may thus have increased of brain activity after treatment.

In the behavioral testing, castrated animals performed poorly in the EPM test, resulting in lower times in the apparatus. These findings are corroborated by previous studies that have shown that gonadectomized animals tend to have impaired locomotor capacity (52, 53). Alterations in alpha wave amplitude have also been related to failures in the execution and visualization of motor processes, as well as affecting motor information processing (54, 55). Taken together, the findings of the present study indicate that the absence of testosterone (castrated group) induces spatial/motor impairment (reduction in alpha wave amplitude and reduced performance in the EPM test) and that testosterone replacement (castrated + testosterone group) may reestablish important brain connections that are dependent on alpha wave oscillations, which is reflected in an improvement in performance in the EPM test.

The results of the present study also showed that acute exogenous testosterone supplementation had no effect on the lipid profile. However, many previous studies have reported that AASs can cause dyslipidemia, depending on the dose and timing of use (24, 56). Zarei et al. (24) showed that TST at a low dose (20 mg/kg) twice per week over 2 weeks had no effect on plasma cholesterol concentration, but plasma cholesterol concentrations decreased at higher doses (50 mg/kg) of testosterone. The present study found no changes in hepatic or renal function, but a decrease was observed in urea levels in the supplemented castrated group, which was of no clinical significance when considered in isolation. This corroborates the findings of previous studies, which have shown no alteration in renal function following the use of testosterone, with only a slight increase in GOT being observed, suggesting dysfunction in the hepatic metabolism (23).

Overall, then, the results of the study revealed that testosterone supplementation at a high dose are associated with increased electrocorticographic patterns in both lower (delta-theta) and higher (alpha, beta, and gamma) wave frequencies. This indicates that the potential use of testosterone supplementation could cause neurophysiological and psychological disorders. Future studies including cellular and molecular changes will be needed, however, to better elucidate the hormonal mechanisms affecting brain activity.

## Data Availability Statement

The datasets generated for this study are available on request to the corresponding author.

## Ethics Statement

The animal study was reviewed and approved by Ethic Committee on Animal Use of the Federal University of Para.

## Author Contributions

DE, LF, and PB: performed the protocol and drafted the manuscript. MS, GJ, GS, KG, JR, VJ, and EC: performed the protocol and biochemical and hormonal analyses. LF, BM, DL, and MH: analyzed the data and drafted the manuscript. MH and DL: reviewed and edited the manuscript. All authors contributed to manuscript revision, read and approved the submitted version.

### Conflict of Interest

The authors declare that the research was conducted in the absence of any commercial or financial relationships that could be construed as a potential conflict of interest.
